# The Provincial Hospitals' Arrangements

**Published:** 1914-09-05

**Authors:** 


### HOSPITALS AND THE WAR.

### The Provincial Hospitals' Arrangements.

(FROM OUR SPECIAL CORRESPONDENTS.)

### BATH.

The local branch of the Red Cross Society at Keynsham—which is a small town that is situated about seven miles from Bath City—is holding special training classes under Dr. Taylor. The Wesleyan school-room has been offered as a hospital if necessary, and a canvass is being made of the residents in order to obtain the names of those willing to receive convalescent patients. The branch has as its secretary Mrs. Thwaites, who was formerly matron of the Bristol General Hospital, and for its treasurer Mr. J. M. Sheppard, the secretary and house steward of the Royal United Hospital, Bath.

### BRADFORD.

When war seemed imminent four of the resident medical officers of the Royal Infirmary, Bradford, volunteered their services. The resident surgical officer, Mr. Nunneley, was accepted for service under the Admiralty, and is now on duty with His Majesty's hospital ship *Albion*. The house physician, Mr. Magoveny, has received a commission in the R.A.M.C., and is now waiting instructions to take up duty. The pathologist, Mr. Hughes, has joined his Territorial battalion (6th West Yorkshire) on embodiment.

The infirmary has had for some time an agreement with the Admiralty to provide six trained nurses to supplement Queen Alexandra's Royal Naval Nursing Service in time of war. On the declaration of war two sisters were called to Portsmouth on August 4, and two days later four more sisters received instructions to proceed to Grimsby to transform a school into a hospital. This work was accomplished quickly and to the satisfaction of the Naval authorities, and on August 22 they were ordered to the Royal Naval Hospital at Chatham for ward duty.

After consultation, the boards of the Royal Infirmary and the Royal Eye and Ear Hospital have resolved to offer accommodation in these institutions for seventy wounded sailors or soldiers, and the committee of the Woodlands Convalescent Home, at Rawdon, have announced their willingness to provide beds for 130 of the less serious cases. Some Red Cross nurses have been received at the Royal Infirmary and Woodlands Home for training in ward work. The Lord Mayor of Bradford has opened a local fund (which now amounts to nearly £11,000) for the relief of distress arising from the war. In addition to this amount many contributions have been sent from Bradford to the Prince's fund. The Lady Mayoress is head of a band of willing workers who are busy preparing clothing and surgical requirements for the wounded, and ward committees have been formed throughout the city to keep in touch with the dependants of Reservists and Territorials. Several citizens have offered their houses as hospitals.

### LEEDS.

In addition to the base hospital provided by the War Office at the Training College in Beckett's Park, Leeds, where there is possible accommodation for 2,000 patients, and already over 100 patients in residence, the General Infirmary, at the request of the War Office, have promised provision for 115 beds.

The Weekly Board have decided not to reduce the number of their civilian beds, as it was felt that stress caused by the war would increase the present heavy demand for beds. They have therefore decided to equip their out-patient department as a ward, and have made alterations in the fittings of the rooms, installed additional electric light, purchased the necessary bedsteads, bedding, and all other requisites for accommodating the 115 patients, and at three or four hours' notice the department will be fully equipped and ready to accommodate the number promised. To cope with this increased number of patients the nursing staff is being strengthened by the appointment of twenty additional nurses, who will, until the new nurses' home is provided, be accommodated in rooms hitherto set apart for other purposes.

### LEICESTER.

The task of equipping the old County Asylum as a base hospital is being rapidly pushed forward, as previous reports in The Hospital have anticipated, by quite an army of workmen—plumbers, painters, builders, and mechanics of all kinds—under the supervision of Mr. S. P. Pick. Already wards are fitted for the reception of a certain number of wounded and sick soldiers, and the arrangements for nurses' and servants' quarters are well advanced. The kitchen arrangements are also being developed, an *x*-ray plant is in course of installation, and a pathological department, theatre, and dispensary are being provided. Members of the Territorial Nursing Staff have been told to hold themselves in readiness for immediate service.

Six members of the nursing staff of the Leicester Royal Infirmary who volunteered in connection with Queen Alexandra's Imperial Military Nursing Service have been called up for duty and have left. Under the supervision of Mr. A. W. Faire, County Director, great activity is being displayed.

Under the scheme formulated by the War Office for the supplementing of the Territorial Medical Service in the event of invasion offers of houses have been received from many residents. Among these offers are that of General Viscount Downe (Dingley Hall), Mr. G. Murray Smith (Gurnby Hall), Mr. George Green (Market Harborough), the Whitwick Colliery Company, Ltd. (Broomleys), and many others. In every centre in the county classes are being formed in connection with the St. John Ambulance Association and the Red Cross Society, and funds are being raised.

In Leicester itself classes have been formed at the Working Men's College for the instruction of candidates in connection with transport arrangements and the conveyance of wounded and the sick. About 150 men are now receiving instruction in this useful branch of service.

The committee of the Leicestershire Automobile Club have been very active in forwarding to the War Office lists of offered motor-cars. More than 100 cars have been placed on the register for conveying wounded and sick soldiers from the Leicester railway stations to the local hospitals if and when required. In response to an urgent communication from London members have quickly placed at the disposal of the Government for service at short notice on the East Coast double the number of cars asked for.

Following the plan originally proposed, the board of governors of the Leicester Royal Infirmary have, in case of need, arranged to place up to 100 beds at the disposal of the 5th Northern General Hospital authorities for sick and wounded soldiers.

In connection with the mobilisation of the 5th Northern General Hospital, the following members of the honorary staff of the Leicester Royal Infirmary have been called up:—Lieutenant-Colonel Pratt, Major Blakesley, and Captain Cumberlidge. From the resident staff:—Messrs. L. T. Challenor and M. H. Barton have been gazetted to commissions in the North Midland Field Ambulance (R.A.M.C.), and Mr. F. A. V. Denning to a probationary surgeoncy on the hospital ship *Rewa*.

The governors of the Loughborough Hospital and Dispensary have also decided to accommodate a certain number of sick and wounded.

### LINCOLN.

The Territorial base hospital is now completed and ready to receive the 520 patients for which it was intended. Additional accommodation is shortly to be provided, which will enable the hospital to take an additional forty patients. A few patients (not wounded) have already been admitted, and more are expected shortly.

### NORTH DEVON DIVISION.

A meeting was held at Barnstaple on Friday, August 28, to consider the advisability of the two small detachments from the villages of Instow and Braunton combining to prepare a hospital of forty beds in the music-hall, Barnstaple. Colonel O'Brien, R.A.M.C., temporary divisional director, together with Dr. Gibbs, Commandant Barnstaple Detachment, Mrs. Mills, Commandant Instow Detachment, and Miss Besley, Commandant Braunton Detachment, were all present. The suggestion was favourably received, as in the event of patients being sent to Barnstaple it seems more economical to concentrate than to send small parties of sick to different villages.

The house committee of the North Devon Infirmary have decided to admit members of the Red Cross Detachments two or three at a time. The hours of attendance will be 7 to 11 a.m., and 5 to 8 p.m. Each batch is to continue for two weeks.

The honorary secretary of the North Devon Infirmary, Captain R. P. Dunn-Pattison, has been called away on duty. Miss Wright, sister, is the only member of the nursing staff who has left. She is a Territorial nurse, and has joined the 4th Northern. Several of the old nurses from this hospital have joined the T.F.N.S. One of the late sisters has returned as temporary sister in Miss Wright's place. The hospital is fortunate in not being depleted of any of its staff, and also is able to continue the nurses' holidays.

### SHEFFIELD.

The arrangements made in Sheffield for dealing with the sick and wounded from the war are on an extensive scale. Not only are the principal medical institutions of the city placing large facilities at the disposal of the military authorities, but a number of educational buildings have been equipped as hospitals by the Territorial Forces. Sheffield, Leeds, and Newcastle, as noted in The Hospital of August 15 (p. 549), are the three northern cities which will serve as hospital centres in connection with the 3rd Northern General Hospital of the Royal Army Medical Corps. A total of 520 beds is provided in Sheffield, distributed among the King Edward VII. School, the Women's Hostel in Collegiate Crescent, the Men's Training College, and the Men's Hostel in Clarkehouse Road, all buildings belonging to the Education Committee which have been formally taken possession of by the 3rd Northern General Hospital.

Lieut.-Col. Connell is in command, and the war strength of the hospital is twenty-one officers and 109 men, together with a nursing staff of ninety-two, of which the principal matron is Miss Smeeton, matron of the Royal Infirmary. Messrs. Seebohm and Dieckstahl, Limited, steel manufacturers, have offered their new offices and warehouse for use as an arrival hospital.

At the Royal Infirmary 100 beds have been fully equipped for dealing with the wounded. The Convalescent Home at Conisborough has also been entirely thrown open to the military authorities, and can be used either for convalescents or for those who are not seriously wounded. Miss Hall, the matron of the Home, who is a Territorial nurse, will be in charge at Conisborough, together with a portion of the infirmary staff. Twenty-five ladies are attending a course of training in bandaging, nursing, and other work to fit them for duty as temporary nurses, which is being conducted at the infirmary. This course will continue for a month, at the end of which time probably another twenty-five will be trained. A number of Territorial medical men are also going through a course of hospital training at the infirmary.

[*Owing to the great pressure on our space several accounts are unavoidably held over.*—Ed.]

